# Urotensin II-induced store-operated Ca^2+^ entry contributes to glomerular mesangial cell proliferation and extracellular matrix protein production under high glucose conditions

**DOI:** 10.1038/s41598-017-18143-x

**Published:** 2017-12-22

**Authors:** Hitesh Soni, Adebowale Adebiyi

**Affiliations:** Department of Physiology, College of Medicine University of Tennessee Health Science Center, Memphis, TN USA

## Abstract

Glomerular mesangial cell (GMC) proliferation and matrix expansion are pathological hallmarks of a wide range of kidney diseases, including diabetic nephropathy. Although the circulating level of peptide hormone urotensin II (UII) and kidney tissue expression of UII and UII receptors (UTR) are increased in diabetic nephropathy, it remains unclear whether UII regulates GMC growth and extracellular matrix (ECM) accumulation. In this study, we tested the hypothesis that UII-induced Ca^2+^ signaling controls GMC proliferation and ECM production under normal and high glucose conditions. Mouse GMCs cultured under normal glucose conditions proliferated and synthesized ECM proteins in response to stimulation by mouse UII. UII-induced GMC proliferation and ECM protein synthesis were dependent on TRPC4 channel-mediated store-operated Ca^2+^ entry (SOCE) and sequential activation of Ca^2+^/calmodulin-dependent protein kinase II (CaMKII) and Ca^2+^/cAMP response element-binding protein (CREB) transcription factor. Under high glucose conditions, GMCs synthesized UII. Moreover, proliferation and ECM production in high glucose-challenged GMCs were attenuated by selective UTR antagonist, TRPC4 channel blocker, and CaMKII and CREB-binding protein/p300 inhibitors. These findings indicate that UII-induced SOCE via TRPC4 channels stimulates CaMKII/CREB-dependent GMC proliferation and ECM protein production. Our data also suggest that UII synthesis contributes to GMC proliferation and ECM accumulation under high glucose conditions.

## Introduction

Experimental data from a variety of animal models suggest that peptide hormone urotensin II (UII) regulates renal functions, including vascular bed perfusion, glomerular filtration, and electrolyte homeostasis^1–3^. Alterations in UII and UII receptor (UTR) tissue expression and circulating and urinary levels of UII have been reported in human and experimental animals with cardiovascular and renal diseases, including hypertension, renal failure, congestive heart failure, atherosclerosis, renal fibrosis, glomerulonephritis, and diabetes^1,2,4–8^. Plasma and urinary concentrations of UII are elevated in proteinuric and non-proteinuric diabetic patients with progressive loss of renal functions^4,7^. Increased expressions of UII and UTR in kidney specimens from human and animals with diabetic nephropathy have also been reported^5,8^. However, the association between UII signaling and cellular events that underpin diabetic nephropathy is poorly understood.

The pathological hallmarks of diabetic nephropathy include glomerular ultrastructural changes, such as basement membrane thickening, extracellular matrix (ECM) accumulation, and mesangial expansion^9,10^. Increased mesangial expansion leads to encroachment of the Bowman’s space, obstruction of the glomerular capillaries, and progressive impairment of glomerular hemodynamics^9,10^. Exposure of cultured glomerular mesangial cell (GMCs) to high glucose concentrations induces proliferation, ECM protein synthesis, and hypertrophy, thereby mimicking the effect of hyperglycemia in diabetic nephropathy^10,11^. Mechanisms that underlie GMC responses to high glucose conditions are not fully resolved, but may include modulation of intracellular Ca^2+^ ([Ca^2+^]_i_), a major regulator of signaling pathways associated with cell cycle control^12^.

An increase in [Ca^2+^]_i_ concentration can be triggered by an influx of extracellular Ca^2+^ into the cells via plasma membrane-localized Ca^2+^-permeable channels or Ca^2+^ release from the intracellular stores or both. Changes in [Ca^2+^]_i_ is turned into biological responses by regulatory proteins that propagate Ca^2+^-sensitive signal transduction mechanisms such as protein phosphorylation and de-phosphorylation to the nucleus to influence gene transcription^12,13^. Like in many other cell types, Ca^2+^-sensitive transcription factors, such as the nuclear factor kappa-light-chain-enhancer of activated B cells, nuclear factor of activated T-cells, and Ca^2+^/cAMP response element-binding protein (CREB) control GMC survival^14–16^. High glucose stimulates CREB phosphorylation in GMCs^17^. Inhibition of [Ca^2+^]_i_ elevation by Ca^2+^ channel blockers, inhibited proliferation, ECM protein synthesis, and CREB activity in GMCs^14^. Hence, CREB target genes are downstream effectors of Ca^2+^-dependent cellular events that promote GMC proliferation and ECM protein accumulation.

Ca^2+^-permeable ion channels that control glomerular function in health and disease include the transient receptor potential cation channels, subfamily C (TRPC). TRPC channels, comprising of seven members (TRPC1-7) function as Ca^2+^ release channels in excitable and non-excitable cells^18^. These channels contribute to Ca^2+^ signaling in GMCs, including store-operated Ca^2+^ entry (SOCE)^19^. SOCE occurs following endoplasmic reticulum (ER) Ca^2+^ store depletion and succeeding extracellular Ca^2+^ influx via store-operated Ca^2+^ channels^20^. TRPC4 constitutes store-operated Ca^2+^ channels in mouse GMCs^21^. TRPC4 can also interact with other TRPC isoforms and Ca^2+^ sensor stromal interaction molecule-1 (STIM1) to form signaling complexes that regulate SOCE in human GMCs^22,23^. UII-induced SOCE resulted in vascular smooth muscle cell proliferation^24^. Our laboratory has also demonstrated that activation of UTR by UII stimulates SOCE in mouse GMCs^25^. However, it is unclear whether SOCE elicited by UII involves TRPC4 channels and controls GMC growth. Given that both UII production and mesangial expansion are associated with diabetic nephropathy^4,7,9,10^, we tested the hypothesis that UII-induced SOCE via TRPC4 channels modulates mouse GMC growth and ECM protein accumulation under normal and high glucose conditions.

## Results

### TRPC4 channels mediate UII-induced SOCE in mouse GMCs

**Figure 1:**
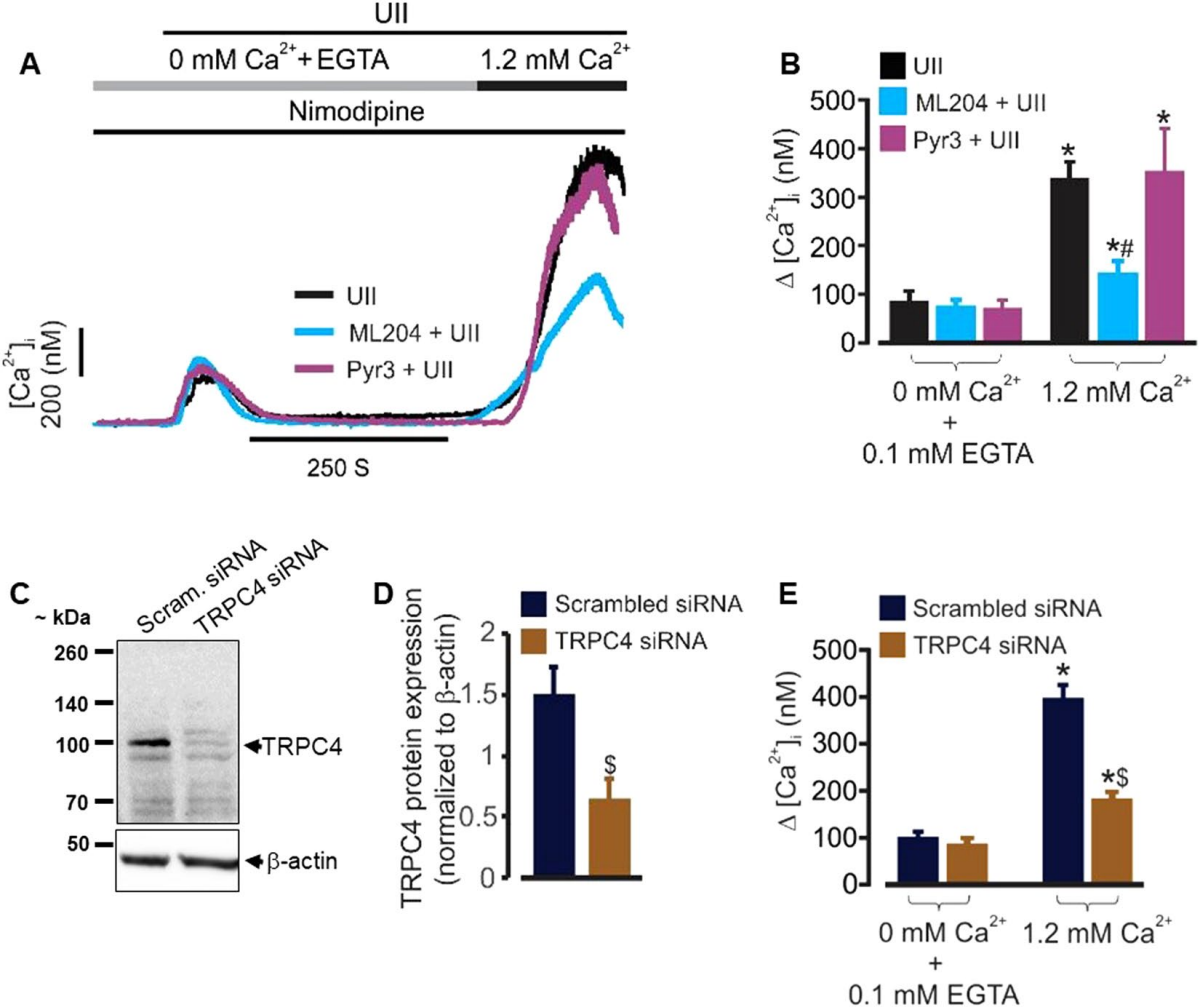
UII stimulates SOCE via TRPC4 channels in mouse GMCs. (**A,B**) Representative traces and mean data showing that UII (100 nM)-induced SOCE is attenuated by ML204 (0.5 µM; n = 7), but not, Pyr3 (1 µM; n = 4). (**C**,**D**) Western blot and mean data (n = 4 each) confirming siRNA-mediated knockdown of TRPC4 channels in mouse GMCs. (**E**) Mean data illustrating that siRNA-mediated knockdown of TRPC4 channels inhibits UII (100 nM)-induced SOCE in mouse GMCs [scrambled (Scram) control siRNA, n = 8; TRPC4 siRNA, n = 7]. The Ca^2+^ free solution was supplemented with EGTA (0.1 mM) to chelate Ca^2+^. To block L-type calcium channels, nimodipine (1 µM) was added to the bath solution. *P < 0.05 vs. 0 mM Ca^2+^ + 0.1 mM EGTA; ^#^P < 0.05 vs. UII and Pyr3 + UII; ^$^P < 0.05 vs TRPC4 siRNA.

To elucidate the role of TRPC4 channels in UII-induced [Ca^2+^]_i_ elevation, we first examined whether ML204, a selective TRPC4 channel blocker^26^ alters UII-induced SOCE in the cells. The effect of Pyr3, a selective TRPC3 channel blocker^27^ on UII-induced SOCE was also examined. In the absence of extracellular Ca^2+^, UII-induced [Ca^2+^]_i_ elevation was unaffected by ML204 and Pyr3 (Fig. 1A and B). However, UII-induced [Ca^2+^]_i_ elevation following extracellular Ca^2+^ re-addition (indicative of SOCE) was attenuated by ML204, but not, Pyr3 (Fig. 1A and B). Next, we measured SOCE in cells transfected with scrambled control and TRPC4 siRNAs. Transfection of GMCs with siRNA targeted to TRPC4 reduced its protein expression (Fig. 1C and D). Accordingly, TRPC4 channel knockdown attenuated UII-induced SOCE in the cells (Fig. 1E). These data indicate that UII-induced SOCE in mouse GMCs is dependent on TRPC4 channels.

### UII-induced SOCE via TRPC4 channels promotes mouse GMC proliferation

**Figure 2:**
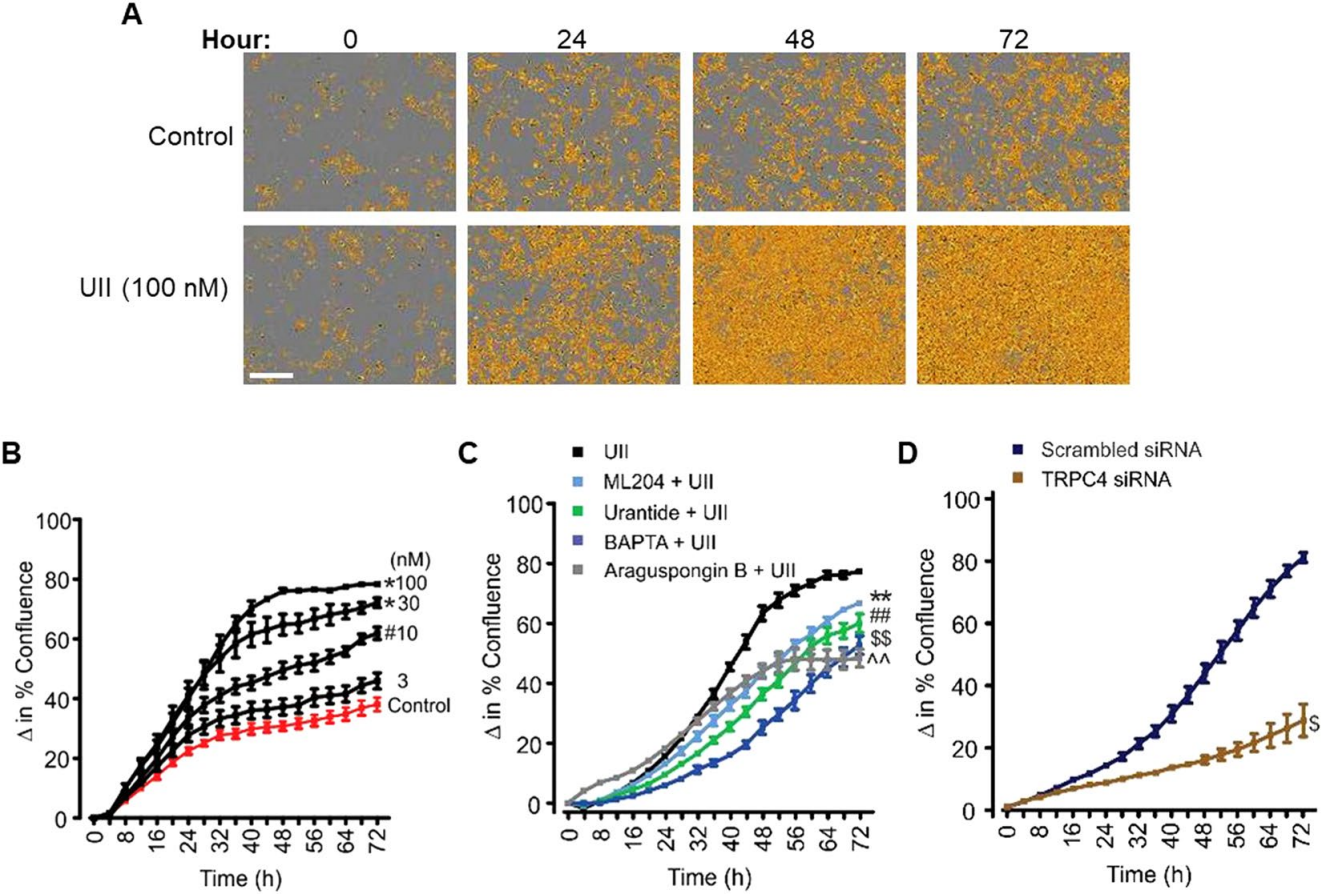
UII-induced SOCE via TRPC4 channels promotes mouse GMC proliferation. (**A)** Phase contrast images demonstrating cell density in control and UII-treated GMCs. (**B)** Cell growth curves showing time- and concentration-dependent proliferative effect of UII (n = 4 each) in mouse GMCs. (**C**,**D)** Cell growth curves indicating that UII (100 nM; n = 4)-induced GMC proliferation is attenuated by urantide (1 µM; n = 5), ML204 (100 nM; n = 5), BAPTA (2 µM; n = 5), araguspongin B (3 µM; n = 5), and siRNA-mediated knockdown of TRPC4 channels (control and TRPC4 siRNA, n = 4 each). “n” for cell proliferation assay denotes number of wells. Each time point represents 4 independent scans per well of the microplates. ^#^P < 0.05 vs. control (24–72 h); *P < 0.05 vs. control (20–72 h); **P < 0.05 vs. UII (34–72 h); ^##^P < 0.05 vs. UII (26–72 h); ^$$^P < 0.05 vs. UII (22–72 h); ^^^^P < 0.05 vs. UII (38–72 h); ^$^P < 0.05 vs. TRPC4 siRNA (32–72 h); Scale bar = 300 µm.

To examine whether UII-induced Ca^2+^ signaling controls GMC growth, cell growth kinetics were assessed in real-time. Cell growth curves automatically quantified over 72 h showed that UII increased GMC confluence in a concentration- and time-dependent manner (Fig. 2A,B). Significant increases in cell confluence commenced ~24 h following UII treatment (Fig. 2A,B). Furthermore, we tested the hypothesis that UTR activation and succeeding SOCE via TRPC4 channels contribute to UII-induced GMC proliferation. Selective UTR antagonist urantide, TRPC4 channel blocker ML204, inositol trisphosphate (IP_3_) receptor antagonist araguspongin B, and [Ca^2+^]_i_ chelator BAPTA, all attenuated UII-induced GMC proliferation (Fig. 2C). Similarly, TRPC4 knockdown diminished the proliferative effect of UII in the cells (Fig. 2D). These results signify that UII-induced SOCE via TRPC4 channels and subsequent [Ca^2+^]_i_ elevation elicit GMC proliferation.

### UII activates Ca^2+^/calmodulin-dependent protein kinase (CaMK) II phosphorylation in mouse GMCs

**Figure 3:**
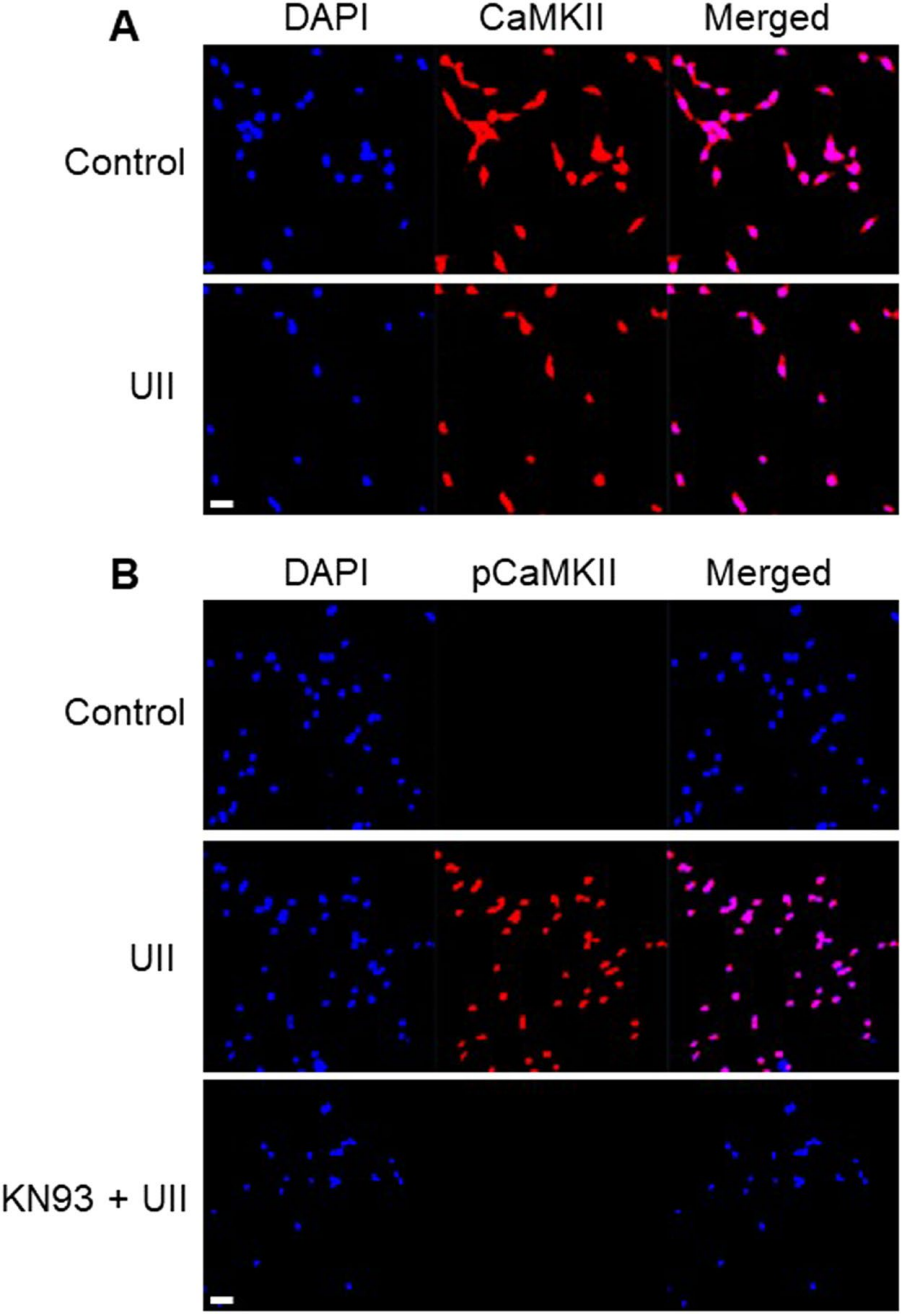
UII activates CaMKII phosphorylation in mouse GMCs. Confocal microscopy of mouse GMCs showing that: (**A)** Total endogenous CaMKII is immunostained in untreated and treated cells (100 nM UII for 5 min), (**B**) Unlike untreated cells, pCaMKII Thr^286^ immunostaining was detected in cells incubated with UII, an effect abolished by KN-93 (2 µM). Images are representative of 3-4 randomly chosen fields from 3-4 coverslips per experimental condition. Scale bar = 100 µm.

To study the mechanisms that underlie UII-induced GMC growth, we examined Ca^2+^-sensitive intracellular signaling proteins that are regulated by UII. First, we investigated whether UII stimulates CaMKII phosphorylation in the cells. CaMKII activation is elicited by binding of Ca^2+^/calmodulin to the regulatory domain of the kinase thereby resulting in phosphorylation at Thr^286^ ^28^. Confocal microscopy demonstrated localization of total endogenous CaMKII in control and cells treated with UII (Fig. 3A). Unlike the untreated cells, phosphorylated CaMKII (pCaMKII; Thr^286^) immunostaining was detected in cells treated with UII, an effect abolished by KN-93, a CaMKII inhibitor (Fig. 3B). These data indicate that UII induces CaMKII phosphorylation in GMCs.

### UII-induced [Ca^2+^]_i_ elevation and CaMKII activation stimulate nuclear pCREB activity in mouse GMCs

**Figure 4:**
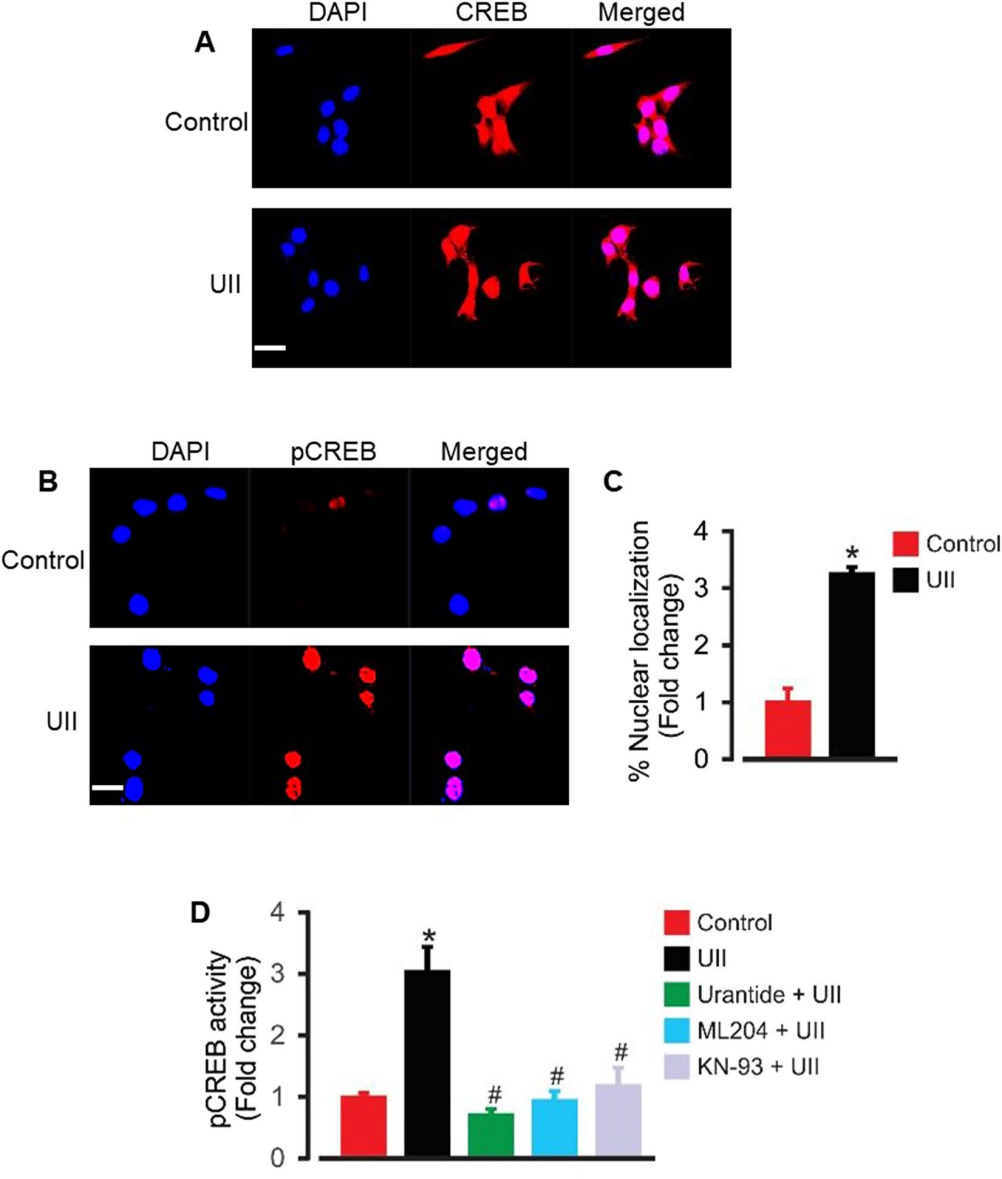
UII-induced [Ca^2+^]_i_ elevation and CaMKII activation stimulates nuclear pCREB activity in mouse GMCs. (**A**,**B**) Confocal microscopy showing localization of endogenous CREB and pCREB in control and UII (100 nM)-treated GMCs. (**C**) Mean data showing that treatment of mouse GMCs with UII (100 nM) for 4 h increased nuclear pCREB immunostaining (n = 20 randomly chosen fields from 3–4 coverslips per experimental condition). (**D**) Mean data from pCREB transcription factor activity assay illustrating that UII (100 nM) increased GMC nuclear pCREB binding activity ~3-fold, an effect inhibited by urantide (1 µM), ML204 (100 nM), and KN-93 (2 µM); n = 5 each. *P < 0.05 vs. control; ^#^P < 0.05 vs. UII. Scale bar = 20 µm.

An elevation in [Ca^2+^]_i_ contributes to the regulation of transcriptional factors that control cell cycle, including CREB^29^. CREB activation is involved in GMC proliferation^14,17^. Given that CREB (at Ser^133^) is a substrate for CaMKII-induced phosphorylation^30^, we investigated the hypothesis that UII-induced [Ca^2+^]_i_ elevation and CaMKII activation stimulate nuclear pCREB activity in GMCs. Immunofluorescence staining showed that endogenous CREB was unchanged in control and cells treated with UII (Fig. 4A). However, UII treatment caused a ~3-fold increase in nuclear pCREB Ser^133^ immunostaining (Fig. 4B and C). Similarly, UII increased pCREB binding activity ~3-fold in GMC nuclear extracts (Fig. 4D). UII-induced nuclear pCREB binding activity was inhibited by urantide, ML204, and KN-93 (Fig. 4D). These data suggest that UII-evoked [Ca^2+^]_i_ elevation and CaMKII induction promote pCREB activation in GMCs.

### UII-induced GMC proliferation is dependent on CaMKII activation and CREB coactivators CREB binding protein (CBP) and E1A binding protein p300 (p300)

**Figure 5:**
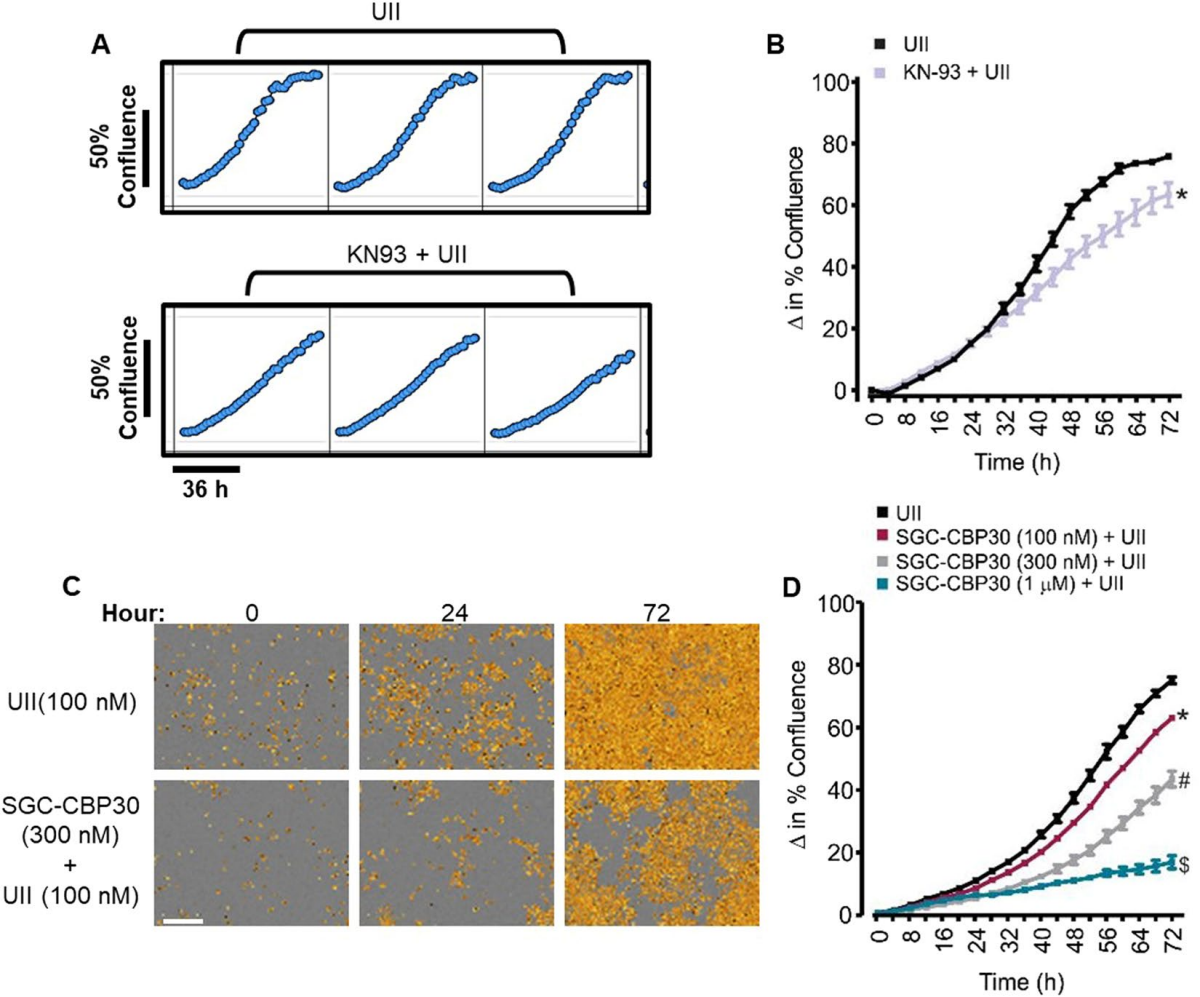
UII-induced GMC proliferation is dependent on CaMKII activation and CREB co-activators CBP/p300. (**A**) Microplate graphs, and (**B)** Cell growth curves demonstrating that UII (100 nM; n = 5)-induced mouse GMC proliferation is inhibited by KN-93 (2 µM; n = 5). (**C**) Phase contrast images, and (**D**) Cell growth curves indicating that inhibition of CREB co-activators CBP and p300 by SGC-CBP30 diminished UII (100 nM)-induced GMC proliferation (n = 4 each). “n” for cell proliferation assay denotes the number of wells. Each time point represents four independent scans per well of the microplates. *P < 0.05 vs. control (40-72 h); ^#^P < 0.05 vs. control (22–72 hr); ^$^P < 0.05 vs. control (26–72 hr). Scale bar = 300 µm.

To study the role of CaMKII and CREB in UII-induced GMC proliferation, we examined cell growth in GMCs pretreated with a CaMKII inhibitor. As shown in Fig. 5A and B, KN-93 significantly reduced UII-induced GMC proliferation. Induction of CREB target genes involves the recruitment of CREB coactivators CBP and p300^31,32^. Hence, we explored the effect of SGC-CBP30, a selective CBP/p300 inhibitor^33,34^ on UII-induced GMC growth. SGC-CBP30 concentration-dependently attenuated UII-induced GMC proliferation (Fig. 5C,D). These findings suggest the involvement of CaMKII activation and CREB co-activators in GMC growth elicited by UII.

### UII-induced SOCE and CaMKII/CREB activation increase type IV collagen and fibronectin production in mouse GMCs

**Figure 6:**
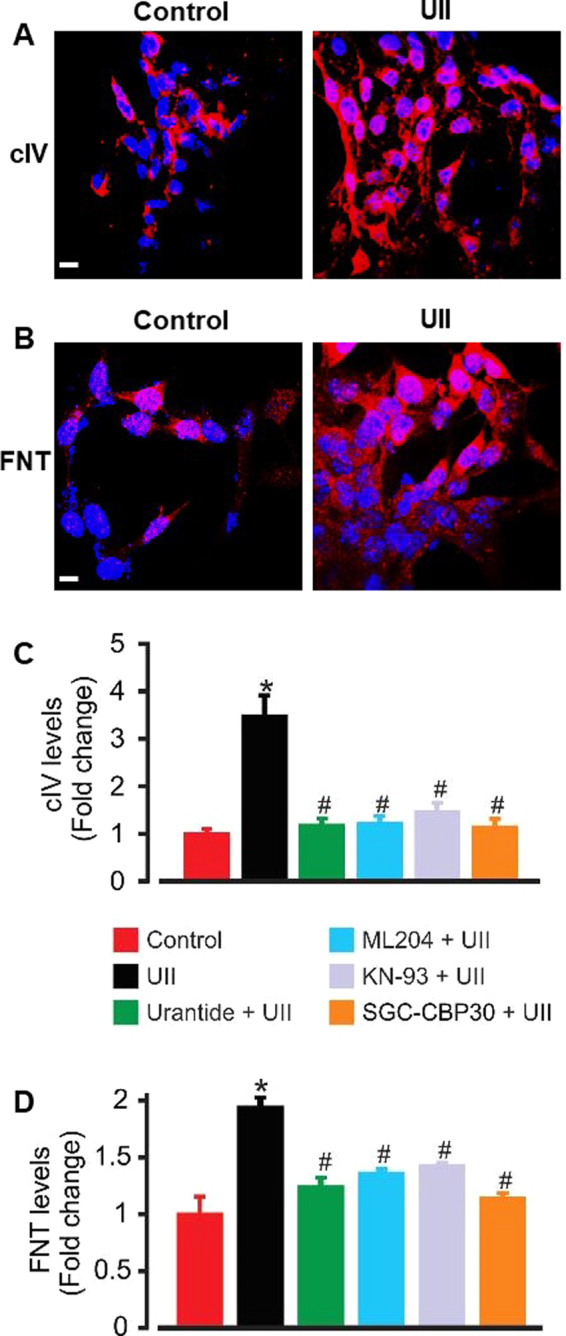
UII-induced Ca^2+^ and CaMKII/CREB signaling increases type IV collagen (cIV) and fibronectin (FNT) accumulation in mouse GMCs. (**A**,**B**) ECM proteins cIV and FNT staining showed higher levels of the proteins in UII (100 nM; 72 h)-treated cells when compared with untreated cells (Images are representative of 3–4 coverslips). (**C**,**D**) Mean data obtained from ELISA illustrating that cIV and FNT productions are increased ~3- and 2-folds, respectively in supernatants of UII (100 nM)-treated GMCs, and that UII-induced cIV and FNT syntheses are inhibited by urantide (1 µM), ML204 (100 nM), KN-93 (2 µM), and SGC-CBP30 (1 µM). For FNT, n = 5 per experimental condition. For cIV, n = 6 (control, urantide, ML204, KN-93, and SGC-CBP30), and 12 (UII). ^*^P < 0.05 vs. control; ^#^P < 0.05 vs. UII.

Next, we investigated the hypothesis that UII stimulates ECM protein synthesis in GMCs. As shown in Fig. 6A and B, GMCs that were immunostained for type IV collagen and fibronectin showed higher levels of the proteins in UII-treated (72 h) cells when compared with untreated cells. Also, type IV collagen and fibronectin concentrations in cell culture supernatants were increased ~3- and 2-fold, respectively in cells treated with UII for 72 h (Fig. 6C,D). UII-induced type IV collagen and fibronectin syntheses were inhibited by urantide, ML204, KN-93, and SGC-CBP30 (Fig. 6C and D). These data indicate that UII-induced SOCE and CaMKII/CREB activation increases ECM protein production in GMCs.

### High glucose-induced UII synthesis promotes mouse GMC proliferation

**Figure 7:**
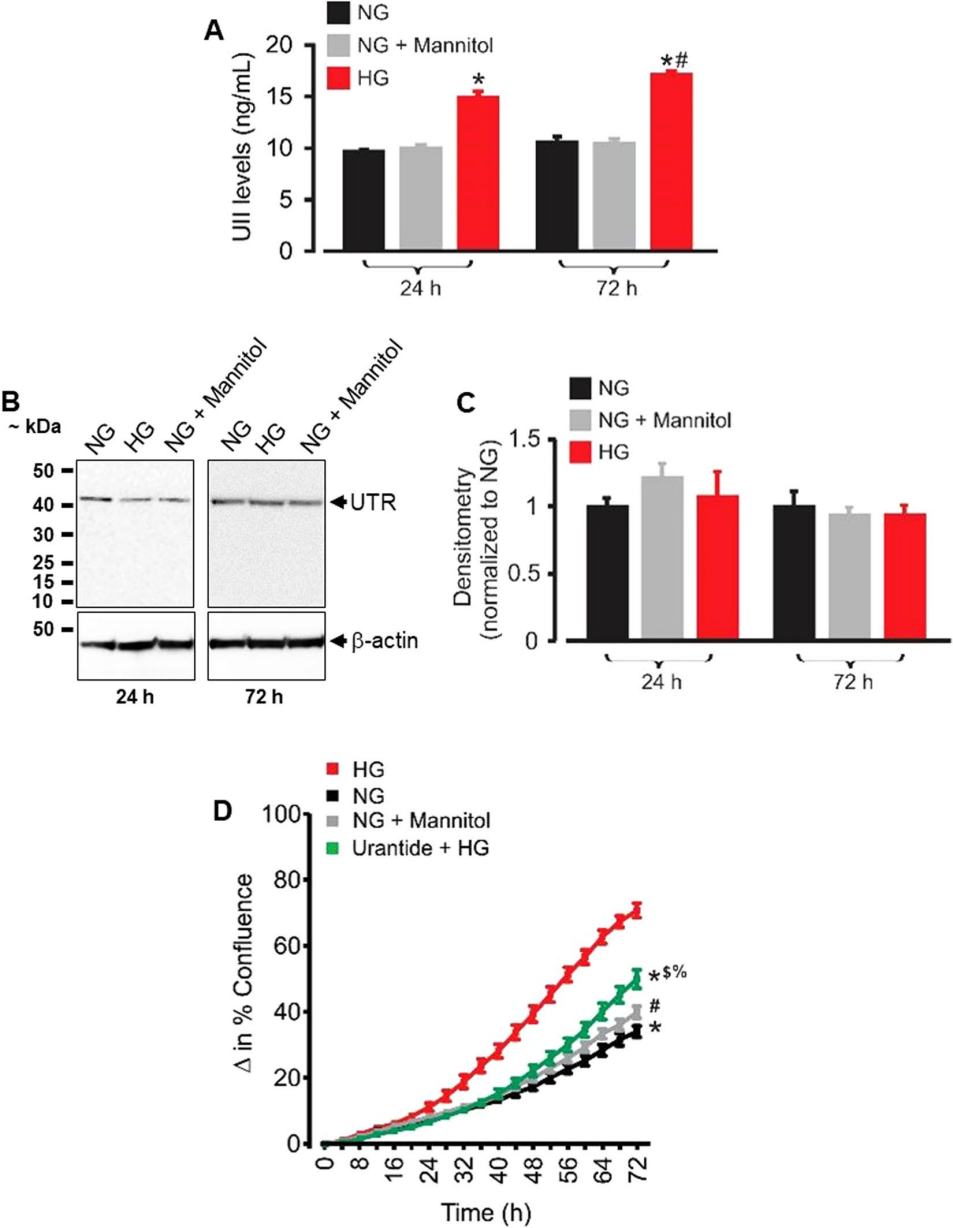
High glucose-induced UII synthesis contributes to GMC proliferation. (**A**) Mean data obtained from mouse UII ELISA showing that exposure of mouse GMCs to high glucose for 24 (n = 6 each) and 72 (n = 9 each) h increases UII production. (**B**,**C**) Western blot and mean data illustrating that exposure of mouse GMCs to high glucose for 24 and 72 h (n = 5 each) does not alter UTR protein expression. (**D**) Cell growth curves demonstrating GMC proliferation under normal glucose (n = 6), normal glucose + mannitol (n = 6), high glucose (n = 6) and urantide (1 µM) + high glucose (n = 6). “n” for cell proliferation assay denotes number of wells. Each time point represents four independent scans per well of the microplates. ^*^P < 0.05 vs. HG (30–72 h); ^#^P < 0.05 vs. HG; (32–72 h); ^$^P < 0.05 vs. NG (54–72 h); ^%^P < 0.05 vs. NG + mannitol (62–72 h). HG (high glucose); NG (normal glucose).

Since UII levels and UTR expression are increased in diabetic nephropathy^4,5,7,8^, it is likely that GMC proliferation under hyperglycemic conditions is associated with the UII/UTR system. To investigate this concept, we first used a mouse UII ELISA kit to measure the concentration of UII in supernatants of GMCs cultured for 24 and 72 h in high (25 mM) glucose DMEM to mimic hyperglycemic conditions typical of diabetic mice^35,36^. As an osmotic control, normal (5.5 mM) glucose-containing DMEM was supplemented with 19.5 mM mannitol. As shown in Fig. 7A, exposure of the cells to high glucose DMEM for 24 and 72 h increased UII concentration in the culture supernatants by ~35 and 39%, respectively. By contrast, UII levels were unchanged in cells cultured in osmotic control medium (Fig. 7A). Western blot analysis indicated that UTR protein expression is unaltered in GMCs that were cultured in high glucose DMEM for 24–72 h (Fig. 7B and C). Unlike the osmotic control, high glucose increased GMC proliferation in a time-dependent fashion (Fig. 7D). Remarkably, high glucose-induced GMC proliferation was attenuated by UTR antagonist urantide (Fig. 7D). These results suggest that an increase in UII production under high glucose conditions, and the resultant activation of UTR induces GMC proliferation.

### TRPC4-dependent [Ca^2+^]_i_ elevation and CaMKII/CREB activation contribute to high glucose-induced mouse GMC proliferation

**Figure 8:**
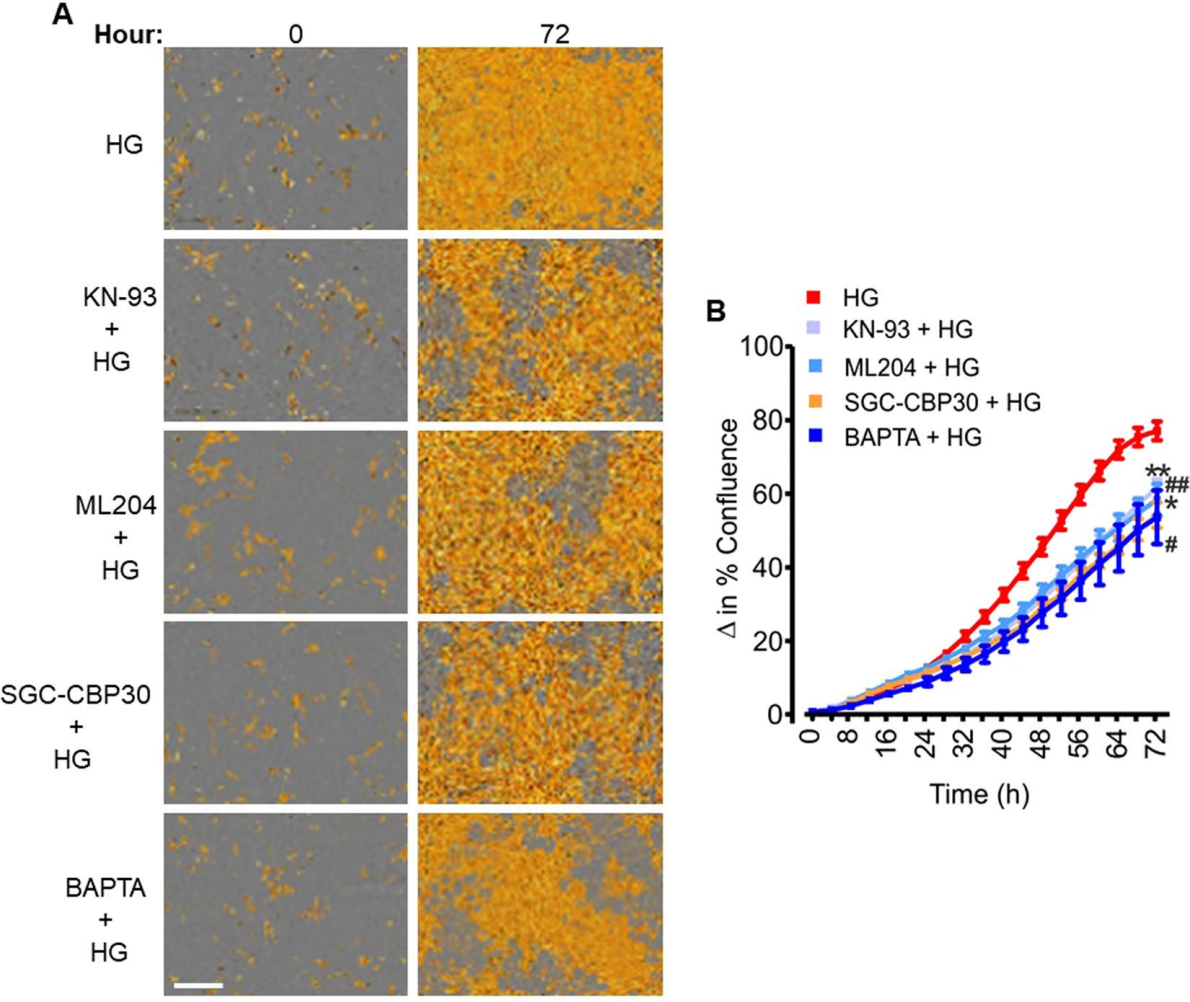
TRPC4-dependent [Ca^2+^]_i_ elevation and CaMKII/CREB activation contribute to high glucose-induced GMC proliferation. (**A**) Phase contrast images, and (**B**) Cell growth curves showing that high glucose-induced mouse GMC proliferation is reduced by ML204 (100 nM), BAPTA (2 µM), KN-93 (2 µM), and SGC-CBP30 (1 µM). n = 10 each; “n” denotes the number of wells. Each time point represents four independent scans per well of the microplates. **P < 0.05 vs. HG (42–72 h); ^##^P < 0.05 vs. HG (44–72 hr); *P < 0.05 vs. HG (38–72 hr); ^#^P < 0.05 vs. HG (36–72 hr); Scale bar = 300 µm. HG (high glucose); NG (normal glucose).

To examine whether TRPC4-dependent [Ca^2+^]_i_ elevation and CaMKII/CREB activation are involved in high glucose-induced GMC proliferation, we studied GMC growth in cells pretreated with a TRPC4 channel blocker and inhibitors of CaMKII and CREB coactivators. High glucose-induced GMC proliferation was attenuated by ML204, KN-93, SGC-CBP30, and BAPTA (Fig. 8A and B), indicating that TRPC4-dependent [Ca^2+^]_i_ elevation and CaMKII/CREB activation contribute to high glucose-induced GMC proliferation.

### UII-induced [Ca^2+^]_i_ elevation and CaMKII/CREB activation contribute to ECM accumulation under high glucose conditions

**Figure 9:**
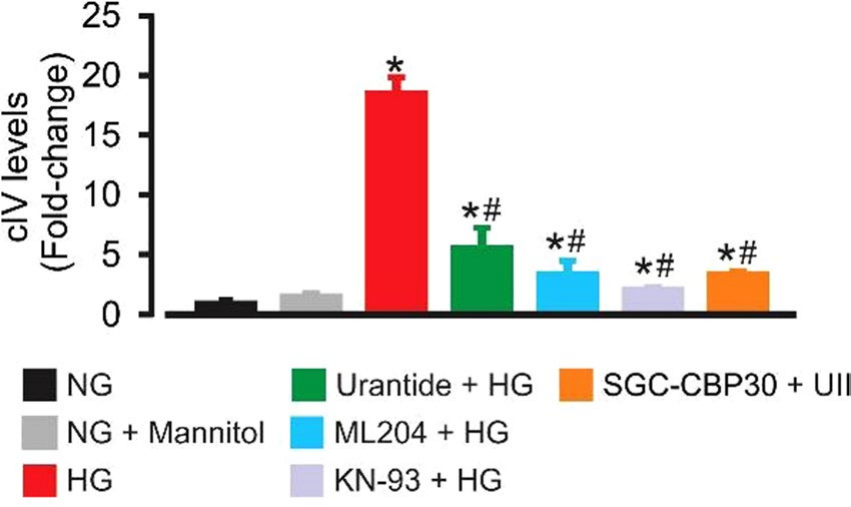
UII-induced [Ca^2+^]_i_ elevation and CaMKII/CREB activation contribute to ECM protein production under high glucose conditions. Mean data obtained from ELISA showing that high glucose-induced type IV collagen production is mitigated by urantide (1 µM), ML204 (100 nM), KN-93 (2 µM), and SGC-CBP30 (1 µM). n = 6 (NG, NG + mannitol, ML204 + HG, KN-93 + HG, and SGC-CBP30 + HG) n = 10 (HG and urantide + HG); *P < 0.05 vs. NG and NG + mannitol; ^#^P < 0.05 vs. HG. HG (high glucose); NG (normal glucose); type IV collagen (cIV).

**Figure 10:**
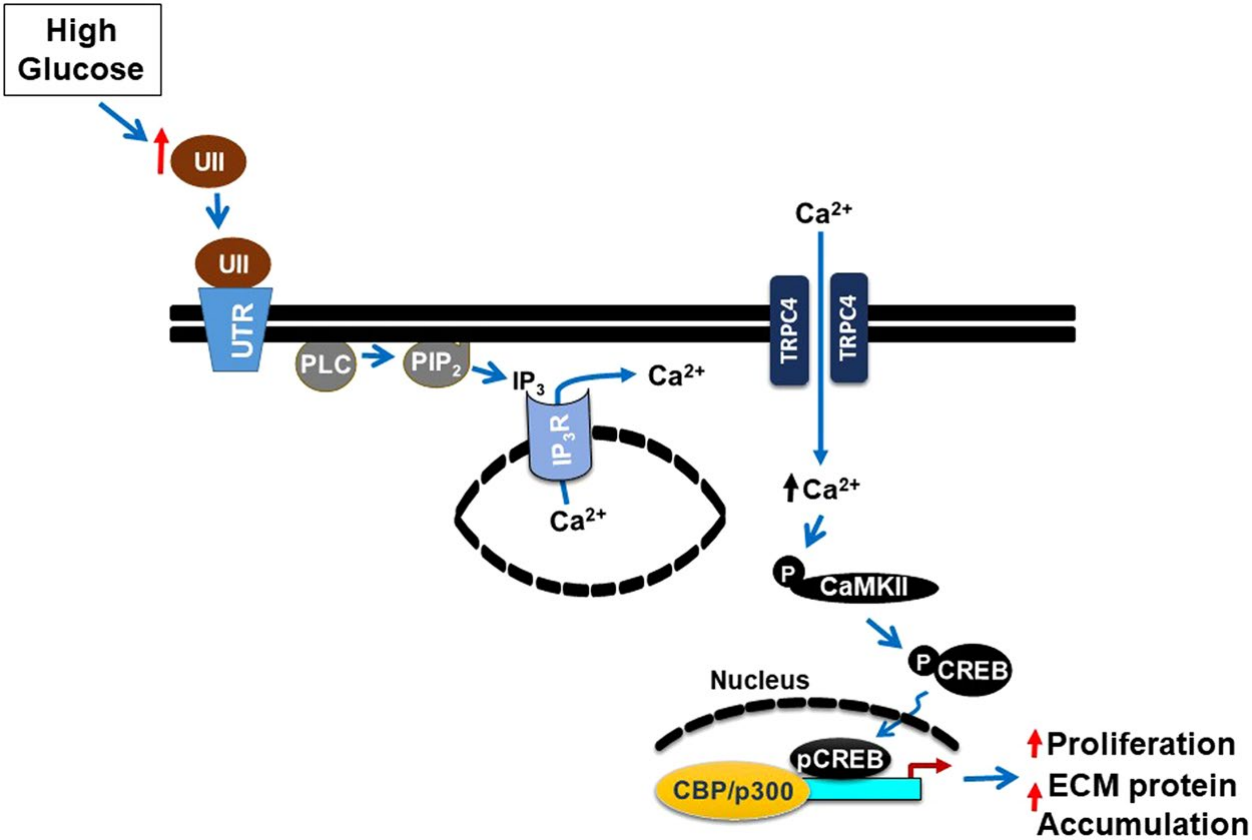
Schematic diagram illustrating hypothetical mechanisms by which UII-induced [Ca^2+^]_i_ elevation via TRPC4 channels, and successive activation of CaMKII/CREB signaling pathways contribute to proliferation and ECM protein accumulation in high glucose-challenged GMCs. Other molecular components of store-operated Ca^2+^ entry, including STIM1 and Orai1 may contribute to these mechanisms.

High glucose increased type IV collagen level in GMC culture supernatants ~19-fold (Fig. 9). High glucose-induced type IV collagen production was attenuated in cells pretreated with urantide, ML204, KN-93, and SGC-CBP30 (Fig. 9). Collectively, our data suggest that UII-induced [Ca^2+^]_i_ elevation via TRPC4 channels, and successive activation of the CaMKII/CREB signal transduction pathway contribute to proliferation and ECM protein synthesis in high glucose-challenged GMCs (Fig. 10).

## Discussion

Although studies have shown that renal UII production may be associated with diabetic nephropathy^1,4,5,7^, little is known about the function of UII in renal glomerulus in health and disease. The findings presented in this study are the first indication that UII-induced [Ca^2+^]_i_ elevation stimulates GMC proliferation and ECM protein production. Our data also suggest that UII is synthesized by GMCs under high glucose conditions, and that high glucose-induced GMC proliferation and ECM protein accumulation are dependent, at least in part, on UII-mediated Ca^2+^ signaling. We thus provide a novel insight into the cellular mechanisms that may contribute to glomerular dysregulation caused by hyperglycemia.

A previous study reported that TRPC4 channel knockdown attenuated SOCE in mouse GMCs by ~83%, indicating that TRPC4 forms store-operated Ca^2+^ channels in the cells^21^. UTR, a Gq/11 protein-coupled receptor is expressed in mouse GMCs^25^. Activation of UTR by UII in mouse GMCs stimulated phospholipase C/ IP_3_-dependent ER Ca^2+^ release, resulting in SOCE^25^. Whether TRPC4 mediates UII-induced SOCE in mouse GMCs was unknown. To delineate the role of TRPC4 in UII-induced SOCE we first studied the effect of ML204, its selective pore blocker^26^. We show that unlike TRPC3 channel blocker Pyr3, a nanomolar concentration of ML204 attenuated UII-induced SOCE. Likewise, SOCE triggered by UII was reduced in cells transfected with TRPC4 siRNA. In rat podocytes, ML204 stimulated Ca^2+^ release from the intracellular stores, but this effect occurred at a concentration 40 times higher than what we used in this study^37^. ML204 can also block TRPC5 channels, albeit at higher concentrations compared with TRPC4^26^. Moreover, TRPC5 is not expressed in mouse GMCs^21^. Hence, the effects of ML204 reported here are independent of TRPC5 channels. Together, our data demonstrate that activation of UTR by UII triggers SOCE via TRPC4 channels, resulting in an increase in [Ca^2+^]_i_ concentration in mouse GMCs. UII-induced SOCE in rat coronary artery smooth muscle cells is mediated by STIM1 and Ca^2+^ release-activated calcium channel (Orai1) protein complex^38^. Similarly, STIM1, Orai1, and TRPC1 contribute to UII-induced SOCE in aortic smooth muscle cells^24^. In human GMCs, SOCE is dependent on protein complexes, including TRPC1, 4, and STIM1^22^. Whether UII-induced TRPC4 activation in mouse GMCs involve its association with TRPC1, STIM1, or Orai1 is not addressed in this study and requires further investigation.

Our data show that CaMKII and CREB phosphorylation are sequential downstream targets of TRPC4-mediated [Ca^2+^]_i_ elevation stimulated by UII in mouse GMCs. Activation of CaMKII and CREB phosphorylation is required for UII-induced vascular smooth muscle cell proliferation^24^. Since UII stimulates CaMKII and CREB phosphorylation via TRPC4-mediated SOCE in mouse GMCs, we investigated the concept that UII-induced GMC proliferation involves CaMKII/CREB activation. We show that at the concentration that prevented UII-induced CaMKII phosphorylation, KN-93 attenuated UII-induced nuclear pCREB binding activity and proliferation in the cells. CREB, a ubiquitous transcription factor controls a wide variety of cellular processes, including GMC survival and ECM protein synthesis^14,39^. Phosphorylation of CREB on Ser^133^ stimulates its interaction with coactivators CBP or its homolog p300, leading to target gene activation^31,32^. CBP/p300 induction promotes cellular apoptosis, proliferation, and development^40^. In rat GMCs, angiotensin II-induced fibronectin upregulation involves activation of CREB and p300^41^. Here, we show that pharmacological inhibition of the CBP/p300 bromodomain^33,34^ suppressed GMC growth in UII-treated cells. These data suggest the involvement of CBP/p300-dependent gene transcription in UII-induced GMC proliferation. Corresponding to its effect on cell number, UII increased type IV collagen and fibronectin secretion by GMCs. Immunofluorescence staining of type IV collagen and fibronectin also indicated that UII stimulates their production by individual cells. These data are consistent with studies that reported an increase in fibronectin and collagen mRNA expression in rat proximal tubular epithelial cells and cardiac fibroblasts treated with UII^42,43^. Thus, a combination of increased proliferation and synthesis by individual GMCs may account for UII-induced ECM accumulation. Our data suggest that UII-induced ECM protein production is dependent on TRPC4 channels, CaMKII, and CBP/p300. Together, our findings highlight the contribution of TRPC4-mediated SOCE and CaMKII/CREB activation to increased growth and ECM production in UII-treated GMCs. CBP/p300 can interact with other transcription factors, such as members of the activator protein 1 (AP-1) family, including c-Fos and c-Jun^40^. Decoy oligodeoxynucleotides targeting AP-1 suppressed high glucose- and angiotensin II-induced rat GMC proliferation and matrix gene expression^44^. Other studies have demonstrated the importance of c-Fos and c-Jun in GMC proliferation^45,46^. Given the molecular and functional crosstalk between CREB and AP-1 signaling pathways^39,47^, UII-induced CREB activation and downstream GMC proliferation and ECM accumulation may involve AP-1 components.

We present novel findings indicating that mouse GMCs synthesize UII when cultured under high glucose conditions. Mechanisms by which high glucose stimulates UII production is unclear but may involve increased proteolytic processing or expression of UII precursor prepro-UII. However, this speculation requires further investigations. Exposure of cultured GMCs to high glucose increases proliferation and accumulation of ECM proteins, especially, type IV collagen and fibronectin, thereby mimicking phenotypic changes in diabetic glomeruli^9–11^. Data here suggest that GMC proliferation and ECM protein accumulation triggered by a high glucose concentration are dependent on UTR, TRPC4 channels, and CaMKII/CREB pathways. These findings are significant because the circulating level and kidney expression of UII are increased in human diabetic nephropathy^4,5,7^. Thus, increased production of UII in diabetes may contribute to mesangial expansion and glomerular derangement. UTR expression in the kidney is upregulated in human and animals with diabetic nephropathy^5,8^. However, we did not observe an increase in UTR protein expression in GMCs exposed to high glucose for 24 and 72 h. Apart from GMCs, UTR is expressed in other locations in the kidney, including collecting ducts, proximal and distal tubules, and blood vessels^1,3^. Hence, other renal cell types may contribute to the increased kidney tissue expression of UTR in diabetes. It is also possible that prolonged exposure of cultured GMCs to high glucose is required to alter UTR protein expression.

Blockade of TRPC4 channels inhibited UII-induced SOCE, CREB activation, proliferation, and ECM production in GMCs. Similarly, TRPC4 channel inhibition attenuated high glucose-induced GMC growth and type IV collagen synthesis. Together, these data indicate that SOCE stimulated by UII contributes to high glucose-induced GMC proliferation and ECM protein accumulation. A recent study reported that activation of SOCE by thapsigargin abolished fibronectin and type IV collagen protein expression in human GMCs cultured in a high glucose medium, suggesting that SOCE elicited by thapsigargin negates ECM accumulation in GMCs^48^. However, unlike UII, thapsigargin-induced [Ca^2+^]_i_ elevation inhibited proliferation and triggered apoptosis in GMCs^49^. The intracellular effectors of thapsigargin-induced SOCE in GMCs are unclear. Thus, it is likely that the effects of SOCE-mediated signal transductions on GMC survival and ECM protein production are dependent on the stimulant, the level of [Ca^2+^]_i_ increase, or downstream Ca^2+^ sensitive signal transduction cascades that are activated. Acute exposure of rat GMCs to high glucose increased [Ca^2+^]_i_ concentration by a mechanism that partly involves the store-operated Ca^2+^ channels^50^. Cyclopiazonic acid- and IP_3_- induced SOCE and store-operated Ca^2+^ currents, respectively have also been shown to be amplified in human GMCs under chronic high glucose treatment, and associated with increased protein expression of STIM1 and Orai1^51^. These studies suggest that additional mechanisms of high-glucose-induced modulation of GMC function may involve direct alterations in basal [Ca^2+^]_i_ levels or expression of the molecular components of the store-operated Ca^2+^ channels.

In conclusion, we present new findings demonstrating that UII-induced Ca^2+^ signaling promotes CaMKII/CREB-dependent proliferation and ECM protein production in GMCs. Our data also suggest that high glucose elicits UII synthesis, which in turn induces CaMKII/CREB-dependent proliferation and ECM protein production in the cells. Conceivably, increased UII production in GMCs and the resultant proliferation and ECM protein accumulation are involved in the pathophysiological mechanisms that underlie mesangial expansion in diabetic nephropathy.

## Methods

### GMC culture

The use of GMC lines was approved, and experiments were performed in accordance with the guidelines and regulations of the Institutional Biosafety Committee of the University of Tennessee Health Science Center. Mouse GMC line (CRL-1927) was purchased from the American Type Culture Collection (Manassas, VA). The cells were subcultured in Dulbecco’s modified Eagle’s medium (DMEM) supplemented with 5% fetal bovine serum and 1% penicillin/streptomycin.

### [Ca^2+^]_i_ imaging

[Ca^2+^]_i_ concentration was measured in GMCs using the ratiometric Ca^2+^ indicator Fura-2 AM, as we have previously done^25,52,53^. Briefly, mouse GMCs cultured in glass-bottom dishes were loaded with Fura-2 AM (10 μM) and 0.5% pluronic F-127 for ~1 h at room temperature. Cells were then washed with modified Krebs’ solution (MKS; 134 mM NaCl, 6 mM KCl, 1.2 mM CaCl_2_, 1 mM MgCl_2_, 10 mM HEPES, and 10 mM glucose, pH 7.4) for ~30 min to de-esterify Fura*-*2 AM molecules. Changes in intracellular Ca^2+^ ([Ca^2+^]_i_) was measured at room temperature with a ratiometric Ca^2+^ imaging system (Ionoptix Corp., Milton, MA, USA). Fura*-*2 AM fluorescence was recorded by using a hyperswitch light source (Ionoptix) to excite at wavelengths of 340 and 380 nm. The fluorescence was collected simultaneously from cells located in one imaging field per dish. Fura-2 AM ratios were background-subtracted and collected at 510 nm using a MyoCam-S CCD digital camera (Ionoptix). Data were then analyzed with the IonWizard software (Ionoptix).

### siRNA transfection

siRNA transfection was performed using the BTX ECM 830 square wave electroporator (BTX, Holliston, MA). Cells (~10^6^) in 100 µl of antibiotic-free DMEM and a pool of 3 target-specific TRPC4 siRNAs (~1 µg) or a non-targeting scrambled control siRNA (Santa Cruz Biotechnology, Inc., Santa Cruz, CA) were electroporated in cuvettes for 100 milliseconds at 150 V. Following recovery (~10 min at room temperature), cells were transferred into flasks or microplates and cultured for 72 hours. Western immunoblotting was used to confirm knockdown efficiency.

### Western immunoblotting

Total protein was isolated from cultured GMCs using the RIPA lysis buffer. Protein concentrations were then quantified using an assay kit and SmartSpec 3000 Spectrophotometer (Bio-Rad, Hercules, CA). Denatured proteins (LDS sample buffer + DTT and heated at 70 °C for 10 min) were separated on polyacrylamide gels in a Mini Trans Blot Cell (Bio-Rad). Proteins were transferred onto nitrocellulose membranes using a Pierce Fast Semi-Dry Blotter (Thermo Scientific). Nonspecific binding sites on the membranes were blocked for ~1 h using Tris Buffered Saline supplemented with 0.05% Tween (TBS-T) and 5% nonfat milk. The membranes were probed with respective primary antibodies overnight at 4 °C. After a wash in TBS-T, the membranes were incubated in HRP-conjugated secondary antibodies for 45 min at room temperature. Immunoreactive proteins were visualized on a Kodak gel imaging system using the femtoLUCENT PLUS-HRP chemiluminescent reagent (G-Biosciences).

### Live content microscopy of GMC growth and immunofluorescence staining

GMC growth was studied using live content microscopy as we have previously described^52,53^. GMCs plated in flat-bottom tissue culture microplates (~1 × 10^4^/well) were starved overnight by culturing in DMEM that contained 0.1% FBS. The cells were then placed in a chamber apparatus of the IncuCyte ZOOM live content microscopy system (Essen Instruments, Ann Arbor, MI) in an incubator. GMC growth was monitored in real-time and quantified from cell confluence changes automatically acquired at two-hourly intervals by the IncuCyte cell density detection software and interface. Phase contrast images (segmentation mask illustrated in goldenrod) demonstrating cell density were also acquired with the IncuCyte software.

For immunofluorescence staining, cells that were sparsely grown on glass coverslips were fixed in 4% formaldehyde for ~20 min and permeabilized with 0.2% Triton X-100 for ~15 min at room temperature. Non-specific binding sites in the cells were blocked for 1 h by incubation in MAXblock blocking medium (Active Motif, Carlsbad, CA). The cells were then treated overnight at 4 °C with respective primary antibodies. Next day, cells were washed with PBS and incubated with cross-adsorbed Alexa Fluor 555 goat anti-rabbit (Life Technologies, Grand Island, NY) for 1 hour at room temperature. Following a wash and mount, fluorescence images were acquired using a Zeiss LSM 710 laser-scanning confocal microscope.

### Phospho-CREB (pCREB; Ser^133^) staining and DNA binding activity

To determine nuclear immunoreactivity of pCREB, the total number of pCREB positive nuclei were counted in randomly-selected microscopic fields and expressed as the percentage of the total number of DAPI-stained nuclei in the same fields. The pCREB transcription factor assay kit (Cayman Chemical, Ann Arbor, MI) was used to detect pCREB activity in GMC nuclear extracts. GMC nuclear proteins were extracted using a nuclear extraction kit (G-Biosciences, St. Louis, MO). pCREB DNA binding activity in nuclear extracts (5 μg protein/well) was determined according to the manufacturer’s instructions.

### Measurement of type IV collagen, fibronectin, and UII production

Cultured GMCs (10^6^/microplate well) were starved overnight. Thereafter, the cells were treated with respective test substances and cultured for 24–72 hours. Type IV collagen, fibronectin, and UII concentrations in cell culture supernatants were measured by enzyme-linked immunosorbent assay (ELISA) kits purchased from Exocell Inc. (Philadelphia, PA), ScienCell Research Laboratories (Carlsbad, CA), and BioTrend Chemicals, LLC (Destin, FL), respectively.

### Antibodies and chemicals

Mouse monoclonal anti-actin and anti-TRPC4 were purchased from Abcam (Cambridge, MA) and Antibodies Inc. (Davis, CA), respectively. Rabbit polyclonal anti-CaMKII, -pCaMKII, and -pCREB were purchased from Cell Signaling Technology (Danvers, MA). Rabbit polyclonal anti-collagen IV and -fibronectin were obtained from Bioss Inc. (Woburn, MA). HRP-conjugated anti-rabbit and anti-mouse secondary antibodies were purchased from Thermo Scientific (Waltham, MA) and Abcam, respectively. Cross-adsorbed Alexa Fluor 555 goat anti-rabbit was purchased from Life Technologies (Grand Island, NY). Fura-2 AM, Pluronic F-127, mouse UII, urantide, β-mercaptoethanol, SGC-CBP30, and Pyr3 were purchased from Life Technologies, AnaSpec (Fremont, CA), Phoenix Pharmaceuticals (Burlingame, CA), Peptides International, Inc. (Louisville, Kentucky), Bio-Rad, Tocris Bioscience (Bristol UK), and Cayman Chemical (Ann Arbor, MI), respectively.

### Data analysis

All data are expressed as mean ± standard error of mean (SEM). Data were analyzed using the InStat software (Graph Pad, Sacramento, CA). Statistical significance was determined by Student’s t-tests for paired or unpaired data and analysis of variance with Bonferroni post-hoc test for multiple comparisons. A *P* value < 0.05 was considered significant.
